# Screening for energetic compounds based on 1,3-dinitrohexahydropyrimidine skeleton and 5-various explosopheres: molecular design and computational study

**DOI:** 10.1038/s41598-020-75281-5

**Published:** 2020-10-26

**Authors:** Binghui Duan, Ning Liu, Xianming Lu, Hongchang Mo, Qian Zhang, Yingzhe Liu, Bozhou Wang

**Affiliations:** 1 Xi’an Modern Chemistry Research Institute, Xi’an, 710065 People’s Republic of China; 2 State Key Laboratory of Fluorine and Nitrogen Chemicals, Xi’an, 710065 People’s Republic of China

**Keywords:** Chemistry, Materials science

## Abstract

In this paper, twelve 1,3-dinitrohexahydropyrimidine-based energetic compounds were designed by introducing various explosopheres into hexahydropyrimidine skeleton. Their geometric and electronic structures, heats of formation (HOFs), energetic performance, thermal stability and impact sensitivity were discussed. It is found that the incorporation of electron-withdrawing groups (–NO_2_, –NHNO_2_, –N_3_, –CH(NO_2_)_2_, –CF(NO_2_)_2_, –C(NO_2_)_3_) improves HOFs of the derivatives and all the substituents contribute to enhancing the densities and detonation properties (*D*, *P*) of the title compounds. Therein, the substitution of –C(NO_2_)_3_ features the best energetic performance with detonation velocity of 9.40 km s^−1^ and detonation pressure of 40.20 GPa. An analysis of the bond dissociation energies suggests that N–NO_2_ bond may be the initial site in the thermal decompositions for most of the derivatives. Besides, –ONO_2_ and –NF_2_ derivatives stand out with lower impact sensitivity. Characters with striking detonation properties (*D* = 8.62 km s^−1^, *P* = 35.08 GPa; *D* = 8.81 km s^−1^, *P* = 34.88 GPa), good thermal stability, and acceptable impact sensitivity (characteristic height *H*_50_ over 34 cm) lead novel compounds 5,5-difluoramine-1,3-dinitrohexahydropyrimidine (K) and 5-fluoro-1,3,5-trinitrohexahydropyrimidine (L) to be very promising energetic materials. This work provides the theoretical molecular design and a reasonable synthetic route of L for further experimental synthesis and testing.

## Introduction

In recent years, energy conversion and storage materials have been a hot area of research in materials science. Metal-ion batteries, solar cells, transition metal dichalcogenides and so on, have significantly enhanced our understanding of hydrogen or solar energy^1–3^. In contrast to batteries and hydrogen-storage materials, energetic materials (EMs) that can store and release a large quantity of chemical energy stand out due to their excellent combustion efficiency and high energy releasing rate. EMs generally referring to explosives, propellants and pyrotechnics are extensively used for military and civilian purposes^4–6^. Modern EMs are expected to have high density, desirable detonation performance and low mechanical sensitivity for safe handling. Recently, hexahydropyrimidine energetic compounds have drawn renewed attention from energetic researchers. With high density, good oxygen balance and low sensitivity, hexahydropyrimidine energetic compounds have displayed potential as energetic additives for high explosives, rocket propellant formulations, and pyrotechnic ingredients^7,8^. 1,3-Dinitrohexahydropyrimidine was firstly synthesized by Bell and Dunstan in 1966^9^ and opened up a new field of cyclic 1,3-dinitramine. Some earlier work has concentrated on the synthesis of nitrohexahydropyrimidine energetic compounds, such as 1,3,5-trinitrohexahydropyrimidine^10^, 1,3,5,5-tetranitrohexahydropyrimidine^11^, 5-azido-1,3,5-trinitrohexahydropyrimidine^12^ and 5,5-difluoramine-1,3-dinitrohexa hydropyrimidine^13^. Although these studies have provided profound insights into the syntheses and thermal decomposition mechanism of hexahydropyrimidine derivatives, there is still lacking comprehensive investigations on explosive performance and systematic molecular design for hexahydropyrimidine compounds. To meet the continuing demand for improved energetic materials, there is a clear need to continue to design and develop new hexahydropyrimidine energetic compounds. This work focus on investigating the most concerned energetic performance, thermal stability and impact sensitivity for 1,3-dinitrohexahydropyrimidine-based energetic compounds that are believed to be candidates of novel EMs. Owing to difficulties in the synthesis of such molecules, theoretical computation is an effective way to design and screen high-energy density compounds.

For EMs, energy and safety are the two most important properties, because energy determines the effectiveness of application and safety guarantees the application^14,15^. The heat of formation (HOF) is frequently used to indicate the “energy content” of an energetic material^16–19^. However, due to the sparsity of experimental data and the lack of systematic theoretical study, HOF values for the title compounds are at present unavailable. It is of considerable importance to estimate HOFs through computational methods. Atomization methods and isodesmic reactions are widely applied to figure out HOFs reliably and rapidly^20,21^. Although these methods often generate some significant errors for various frameworks and groups, the errors are sometimes systematic and can be corrected. As to the thermal decomposition process of EMs, the rupture of “trigger linkage” is believed to be a key factor in detonation initiation^22–24^. Many researchers believe that C–NO_2_, N–NO_2_ and O–NO_2_ bonds are trigger spots in nitro compounds. When it comes to impact sensitivity, Politzer^25^ proposed that it relates to the bond dissociation energy (BDE) of the trigger bond and molecular electrostatic potential (ESP). Bates^26^ suggested that the ability of substitution groups to attract electrons affects the sensitivity of tetrazole: the stronger the ability is, the more sensitive the compound is. Kamlet and Adolph^27^ pointed out that the impact sensitivity of some nitro compounds increases with their enhanced oxygen balance. Zhang advanced a method to assess the impact sensitivity of nitro compounds with Mulliken net charges of nitro groups^28,29^. Accordingly, the above-mentioned methods are referenced to analyze the thermal stability and impact sensitivity of hexahydropyrimidine derivatives and we tried to find an optimal standard to evaluate the impact sensitivity of hexahydropyrimidine compounds.

**Figure 1:**
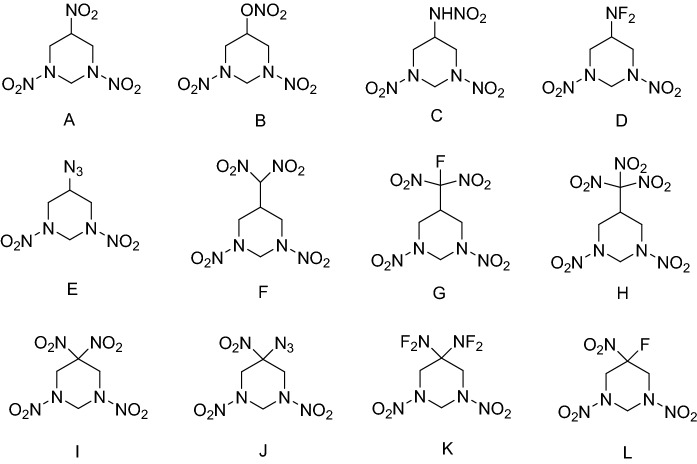
Molecular frameworks of 1,3-dinitrohexahydropyrimidine-based derivatives.

In this paper, we reported a systematic study of the geometric and electronic structures, HOFs, energetic properties, thermal stabilities and impact sensitivities of a series of 1,3-dinitrohexahydropyrimidine-based derivatives (molecular numbering as A ~ L) as shown in Fig. 1. The compounds were designed based on 1,3-dinitrohexahydropyrimidine skeleton and mono- or di-substituted at C5- position. Our main purpose here is to investigate the important role of different substituents in the design of efficient high energetic materials.

## Results and discussion

### Molecular geometry

The optimized structural data for the title compounds are listed in Table 1. Supplementary Figure S1 displays the optimized geometric structures and atomic numbering.

**Table 1:** Optimized bond lengths (Å), bond angles (°) and torsion angles (°) of the title compounds at B3LYP/6-311G(d,p) level of theory.

Geometrical parameter	A	B	C	D	E	F	G	H	I	J	K	L
**Bond length**												
N1–C1	1.449	1.454	1.469	1.446	1.450	1.457	1.466	1.454	1.454	1.467	1.441	1.484
N2–C1	1.479	1.479	1.450	1.480	1.441	1.457	1.463	1.460	1.467	1.466	1.479	1.442
N1–C2	1.461	1.460	1.464	1.462	1.465	1.462	1.459	1.463	1.459	1.457	1.455	1.460
N2–C4	1.461	1.460	1.468	1.458	1.468	1.472	1.460	1.468	1.467	1.461	1.455	1.461
C2–C3	1.548	1.551	1.540	1.553	1.539	1.516	1.549	1.552	1.531	1.542	1.554	1.532
C3–C4	1.536	1.537	1.547	1.540	1.550	1.514	1.552	1.544	1.523	1.544	1.547	1.535
N1–N3	1.425	1.418	1.396	1.421	1.387	1.401	1.395	1.392	1.395	1.400	1.425	1.397
N2–N4	1.381	1.395	1.417	1.384	1.416	1.413	1.394	1.408	1.408	1.400	1.391	1.424
C3–N5	1.529		1.454	1.484	1.472				1.540	1.462	1.505	1.545
C3–N8										1.562		
C3–O5		1.445										
C3–C5						1.336	1.518	1.531				
C5–N5						1.485	1.560	1.562				
**Bond angle**												
C2–N1–N3	115.7	115.6	116.5	116.3	118.7	116.3	118.5	116.8	116.8	118.2	117.2	117.5
C4–N2–N4	119.8	118.1	117.0	119.3	117.7	113.6	118.4	114.3	114.2	118.4	118.4	117.2
C1–N1–N3	116.0	116.5	117.7	116.0	115.2	118.6	115.1	119.1	119.2	114.9	117.2	114.6
C2–C3–N5	111.3		113.1	113.6	112.4				112.3	111.8	113.4	108.5
C3–N6–N5	110.5		119.0									
C2–C3–O5		103.0										
F1–N5–F2				101.8							102.1	
N5–N6–N7					172.2					171.9		
F1–C3–N5							106.9					107.1
C3–C5–N5						123.5	113.5	113.0				
**Torsion angle**												
C1–N1–C2–C3	59.5	− 62.0	− 31.9	− 55.6	− 31.0	37.8	68.7	24.7	32.5	65.0	48.6	− 31.6
C4–N2–C1–N1	− 16.1	14.7	58.3	− 6.9	59.3	30.4	− 35.3	28.5	22.1	− 35.2	12.4	59.7
C2–C3–C4–N2	− 41.5	36.2	− 32.0	40.3	− 29.8	− 54.1	− 22.3	− 65.0	− 59.0	− 32.7	− 44.1	− 28.1
N3–N1–C1–N2	94.7	− 95.5	124.0	− 84.0	− 174.3	146.7	179.8	144.5	144.8	179.3	89.3	− 169.2
N1–C2–C3–N5	112.8		− 177.0	127.4	− 173.9				151.2	94.9	125.8	− 179.4
N1–C2–C3–O5		137.5										
N1–C2–C3–C5						− 160.9	− 156.0	163.6				

As can be seen, the N–N bond lengths in the title compounds range from 1.381 to 1.425 Å, which is shorter than the usual N–N single bond length (1.45 Å). This may be attributed to the hyperconjugation effects between nitro groups and nitrogen atoms. In terms of C–N bonds, some of them in 1,3,5-trinitrohexahydropyrimidine (A), 5-nitrate-1,3-dinitrohexahydropyrimidine (B), 5-fluoramine-1,3-dinitrohexahydro pyrimidine (D), 5,5-difluoramine-1,3-dinitrohexahydropyrimidine (K) and 5-fluoro-1,3,5-trinitrohexahydropyrimidine (L) are longer than normal C–N single bond length (1.472 Å) as a result of cage strain. The substitution of the substituents on hexahydropyrimidine ring should be responsible for some significant difference between the bond lengths and bond angles of the compounds. C3(5)–N5(8) bonds act as the linkage between hexahydropyrimidine ring and the substituents, whose bond lengths are the longest among all the C–N bonds in the molecules except 5-nitramino-1,3-dinitrohexahydropyrimidine (C). It could be deduced that C3(5)–N5(8) bonds are likely to be the trigger bonds in the compounds, which is detailed in the succeeding section.

The C1–N1–C2–C3 and C2–C3–C4–N2 torsion angles for all the hexahydropyrimidine derivatives are larger than 20°, indicating that the planarity of the ring is bad. The distortion of the ring is ascribed to the electron-withdrawing ability of the substituents and the lack of π conjugation interaction in the ring. The torsion angles N3–N1–C1–N2 in A, B, D and K are close to 90°, implying that the –NO_2_ group on N3 are approximately perpendicular to the ring. However, these torsion angles in 5-azido-1,3-dinitrohexahydropyrimidine (E), 5-fluorodinitromethyl-1,3-dinitrohexahydropyrimidine (G), 5-azido-1,3,5-trinitrohexa hydropyrimidine (J) and L are close to 180°, implying a partial planar structure. The torsion angles of the substituents to the ring may be a comprehensive compromise of intermolecular packing and steric hindrance effect.

### Electronic structure

Molecular orbital analysis can provide much useful information on the electronic structure, which is widely used in the chemical reaction. The eigenvalue of the highest occupied molecular orbital (HOMO) characterizes the ability of donating electron and the eigenvalue of the lowest unoccupied molecular orbital (LUMO) characterizes the ability of accepting electron. Energy gap (Δ*E*), the difference between HOMO and LUMO, offers extensive information on the wavelength that a molecule can absorb and indicates the capability of electron transition from occupied orbitals to unoccupied orbitals.

Table 2 lists the calculated HOMO and LUMO energies and the energy gaps (∆*E*_LUMO-HOMO_) for 1,3-dinitrohexahydropyrimidine and its derivatives. When –NO_2_, –ONO_2_, –NHNO_2_, –NF_2_ or –N_3_ group is attached onto the hexahydropyrimidine ring, both of the HOMO and LUMO energies are higher than those of –CH(NO_2_)_2_, –CF(NO_2_)_2_ and –C(NO_2_)_3_ derivatives, and those of –N_3_ derivative are the highest. Additionally, the disubstituted (attached to C atom) compounds exhibit lower HOMO and LUMO energies than corresponding monosubstituted ones unpredictably. The energy gap between HOMO and LUMO relates the kinetic stability, chemical reactivity, and optical polarizability of a molecule. Some investigations on the excitonic mechanism of detonation initiation show that the pressure inside the impact wave front promotes the HOMO → LUMO transition within a molecule^30,31^. The smaller the energy gap, the easier is HOMO → LUMO electron transfer, the shorter wavelength is required for electronic excitation and the easier is explosive decomposition of the energetic material. Thus, 5-trinitromethyl-1,3-dinitrohexahydropyrimidine (H) may be the most reactive and K the least reactive among these compounds. Moreover, it is interesting to note that the incorporation of –NHNO_2_ or –NF_2_ group makes a significant increase of Δ*E* in comparison with the parent compound 1,3-dinitrohexahydropyrimidine (S).

**Table 2:** Calculated HOMO and LUMO energies (a.u.) and energy gaps (Δ*E*, a.u.) of 1,3-dinitrohexahydropyrimidine and its derivatives.

Compd	*E*_HOMO_	*E*_LUMO_	Δ*E*_LUMO-HOMO_
S	− 0.2803	− 0.0671	0.2132
A	− 0.3074	− 0.0994	0.2080
B	− 0.2978	− 0.0919	0.2059
C	− 0.3016	− 0.0864	0.2152
D	− 0.3005	− 0.0878	0.2127
E	− 0.2847	− 0.0814	0.2033
F	− 0.3201	− 0.1481	0.1720
G	− 0.3078	− 0.1335	0.1743
H	− 0.3168	− 0.1514	0.1654
I	− 0.3215	− 0.1348	0.1867
J	− 0.3035	− 0.1108	0.1927
K	− 0.3076	− 0.0903	0.2173
L	− 0.3136	− 0.1139	0.1997

**Figure 2:**
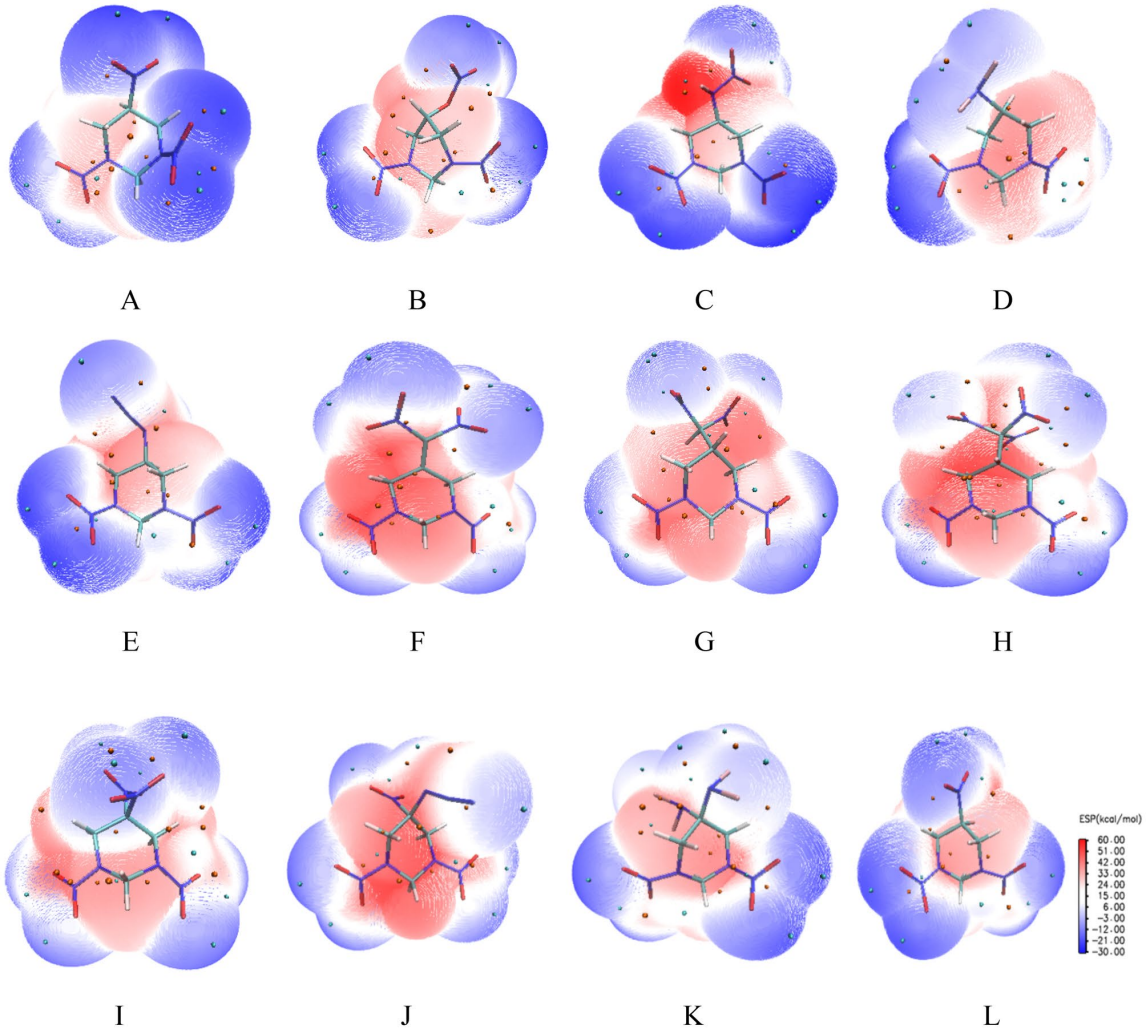
ESP-mapped surfaces of 1,3-dinitrohexahydropyrimidine-based derivatives.

Electrostatic potential (ESP) is a real and fundamentally significant physical property of compounds as it provides information about charge density distribution and molecular reactivity^5,32^. Hence, the surface electrostatic potential was taken into account in the analysis of electronic properties of the title compounds. The ESP-mapped surfaces of 1,3-dinitrohexahydropyrimidine-based derivatives are presented in Fig. 2. The ESPs are scaled with color. Blue denotes the most negative potential (− 30 kcal mol^−1^) and red the most positive potential (60 kcal mol^−1^). Some pivotal maxima and minima of ESPs are expressed by orange and cyan spheres, respectively. It is shown that the strength and orientation of weak interactions can be well predicted and explained by analyzing the magnitude and positions of minima and maxima on the surface. All the ESP surfaces were created with Multiwfn program and visualized with VMD suite^33^.

From Fig. 2, it is clear that the strongly positive ESPs distribute in the central regions of hexahydropyrimidine ring and above N–NO_2_ and C–R bonds, while the negative ones concentrate on the edges of molecules, especially on the oxygen atoms of nitro groups. The charge density distribution and the magnitude of minima and maxima on the surface are definitely affected by the substituent groups. The overlap of positive (red) and negative potential (blue) displays white region and suggests interactions between electron-withdrawing groups and heterocycle. It is interesting to find that most of the linkages (except F, G and H) between hexahydropyrimidine ring and the substituents are colored in white, indicating that the electronic charges tend to be neutral and there may be expected to exist considerable intra- and inter-molecular interactions between hexahydropyrimidine ring and the substituent groups. Studies show that the molecular electrostatic potential is related to the impact sensitivity of the energetic compounds^25^. The electrostatic potential maxima were summarized in Table 4 and they were used to evaluate the impact sensitivity of the title compounds, as detailed in subsequent text.

### Heats of formation

**Figure 3:**
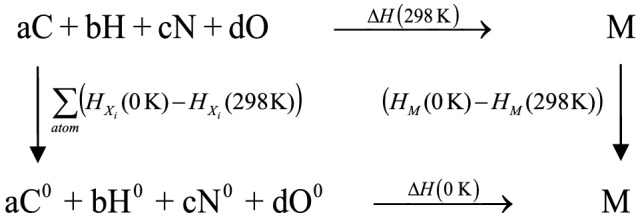
The atomization scheme.

In this paper, atomization approach was adopted to estimate the gas phase heats of formation Δ*H*_f_(g, 298 K). The energy and the enthalpy data needed during the calculation were obtained at G2 level, which has been shown to accurately predict the heats of formation for a variety of organic compounds^34,35^. The detailed calculation procedure is shown as follows (Fig. 3):

The gas state HOF of M at 298 K could be obtained as Eq. (1):

1 $$\Delta H_{f} (g,298K) = \Delta H_{f} (0K) + \sum\limits_{atom} {(H_{{X_{i} }} } (0K) - H_{{X_{i} }} (298K)) + (H_{M} (298K) - H_{M} (0K))$$

where *n*_i_ stands for the number of atoms of *X*_*i*_ in M, *H*_*Xi*_ (0 K) stands for the HOF of *X*_*i*_ at 0 K, which can be found from the NIST-JANAF tables^36^, and Δ*H*(0 K) can be derived from *H*_*Xi*_(0 K). (*H*_*M*_(0 K)–*H*_*M*_(298 K)) and (*H*_*Xi*_(0 K)–*H*_*Xi*_(298 K)) represent the enthalpy correction of the molecule (*M*) and atom (*X*_*i*_) between 0 and 298 K, respectively.

Since the condensed phase for most energetic compounds is solid, the calculation of detonation properties requires solid-phase HOF (Δ*H*_f,solid_). According to Hess’s law of constant heat summation, the gas-phase HOF (Δ*H*_f,gas_) and heat of sublimation (Δ*H*_sub_) can be used to evaluate Δ*H*_f,solid_^37^:

2 $$\Delta H_{f,solid} = \Delta H_{f,gas} - \Delta H_{sub}$$

Politzer et al*.*^38^ found that the heats of sublimation can correlate well with the molecular surface area and electrostatic interaction index *σ*_tot_^2^ of energetic compounds. The empirical expression of the approach is as follows:

3 $$\Delta H_{sub} = \alpha A^{2} + \beta \left( {\nu \sigma_{tot}^{2} } \right)^{0.5} + \gamma$$

where A is the surface area of electronic density of 0.001 electrons/bohr^3^ isosurface of the molecule. The coefficients *α*, *β*, and *γ* were determined by Rice et al.^39^: *α* = 2.670 × 10^–4^ kcal/mol/Å^4^, *β* = 1.650 kcal/mol, and *γ* = 2.966 kcal/mol. This approach has been proved to be credible for evaluating heats of sublimation of many energetic compounds.

**Figure 4:**
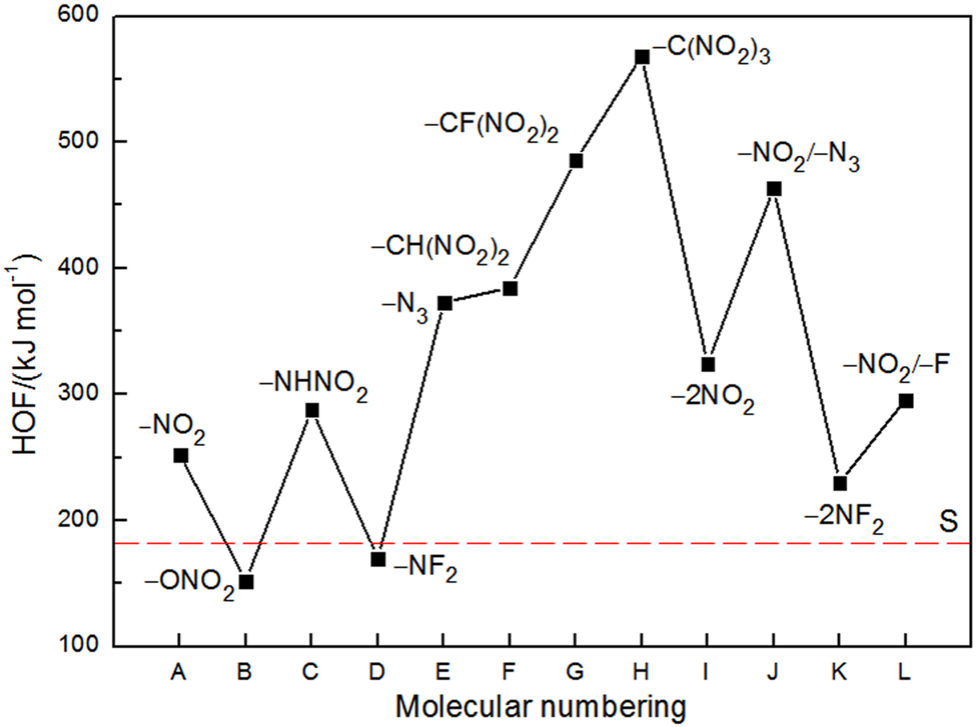
Comparison of HOFs of 1,3-dinitrohexahydropyrimidine-based compounds.

Figure 4 illustrates a comparison of HOFs of the title compounds. Supplementary Table S2 summarizes the calculated gas-phase HOFs (Δ*H*_f,gas_), heats of sublimation (Δ*H*_sub_) and solid-phase HOFs (Δ*H*_f,solid_) of 1,3-dinitrohexahydropyrimidine (S) and its derivatives. As can be seen, all the substituted derivatives possess positive HOFs ranging from 152.173 to 568.252 kJ mol^−1^. When the substituent is –NO_2_, –NHNO_2_, –N_3_, –CH(NO_2_)_2_, –CF(NO_2_)_2_ or –C(NO_2_)_3_ group, the HOF value of its substituted 1,3-dinitrohexahydropyrimidine increases significantly compared with the unsubstituted one (S). Therein, the HOF value of –C(NO_2_)_3_-substituted 1,3-dinitrohexahydropyrimidine (H) is the largest among all the compounds, and this substitution extremely enhances its HOF. However, for the substituent of –ONO_2_ or –NF_2_, the case is the complete opposite. In addition, it is found that the HOF value improves with the increasing number of nitro groups in the substituent, indicating a good group additive effect on the HOFs. It is worthy to note that except for –NO_2_ group, –N_3_ group is also one of the most energetic functional groups known and its substitution can increase the energy content of a molecule greatly. As shown in Fig. 4, all the disubstituted (attached to C atom) compounds exhibit higher HOFs than corresponding monosubstituted ones, implying that increasing the energetic substituents to hexahydropyrimidine ring is favorable to improve the HOF.

### Energetic performance

Density, detonation velocity and detonation pressure are three important parameters reflecting the energetic performance of an energetic material. With a complete neglect of intermolecular interactions within the crystal, conventional *M*/*V* approach to calculate density leads to some large error. An improved method by considering the role played by intermolecular forces in the crystal lattice is shown as follows^40^:

4 $$\rho = \beta_{1} \left( \frac{M}{V} \right) + \beta_{2} \left( {\nu \sigma_{tot}^{2} } \right) + \beta_{3}$$

where *M* is the molecular mass (g mol^−1^), *V* is the volume of the isolated gas molecules defined as the space inside a counter of electron density of 0.001 e Bohr^−3^ using a Monte Carlo integration^41^, *ν* describes the degree of balance between positive and negative potential on the isosurface, and *σ*_tot_^2^ is a measure of variability of the electrostatic potential on the molecular surface. The values of the coefficients *β*_1_, *β*_2_ and *β*_3_ are 0.9183, 0.00278 and 0.0443, respectively. We performed 100 single-point calculations for each optimized structure to get an average volume.

Detonation velocity (*D*) and pressure (*P*) can be estimated using empirical Kamlet-Jacobs equations as follows^42^:

5 $$D = 1.01(NM^{1/2} Q^{1/2} )^{1/2} (1 + 1.30\rho )$$

6 $$P = 1.558\rho^{2} NM^{1/2} Q^{1/2}$$

where *D* is the detonation velocity (km s^−1^), *P* is the detonation pressure (GPa), *N* is the number moles of gaseous products per gram of explosive, *M* is the average molecular weight of gaseous detonation products, *ρ* is crystal density (g cm^−3^) and *Q* is the detonation energy (cal g^−1^). *N*, *M* and *Q* are determined from the stoichiometric reactions developed for maximum exothermic principle, using arbitrary H_2_O–CO_2_–N_2_ decomposition assumption. Due to the explosive and sensitivity nature of the high energy materials, the experimental determination of their *Q* and *ρ* are not very frequent, and theoretical approaches have been found to be a viable option.

Oxygen balance (OB) is used to indicate the degree to which a compound can be oxidized and to classify energetic materials as either oxygen-deficient or oxygen-rich. The higher the oxygen balance is, the larger the detonation velocity and pressure are and the better the performance of the explosive is. For an energetic compound C_a_H_b_O_c_N_d_, OB(CO_2_) was calculated by the following equation^43^:

7 $$OB = 1600 \times (d - 2a - b/2)/M \times 100\%$$

**Figure 5:**
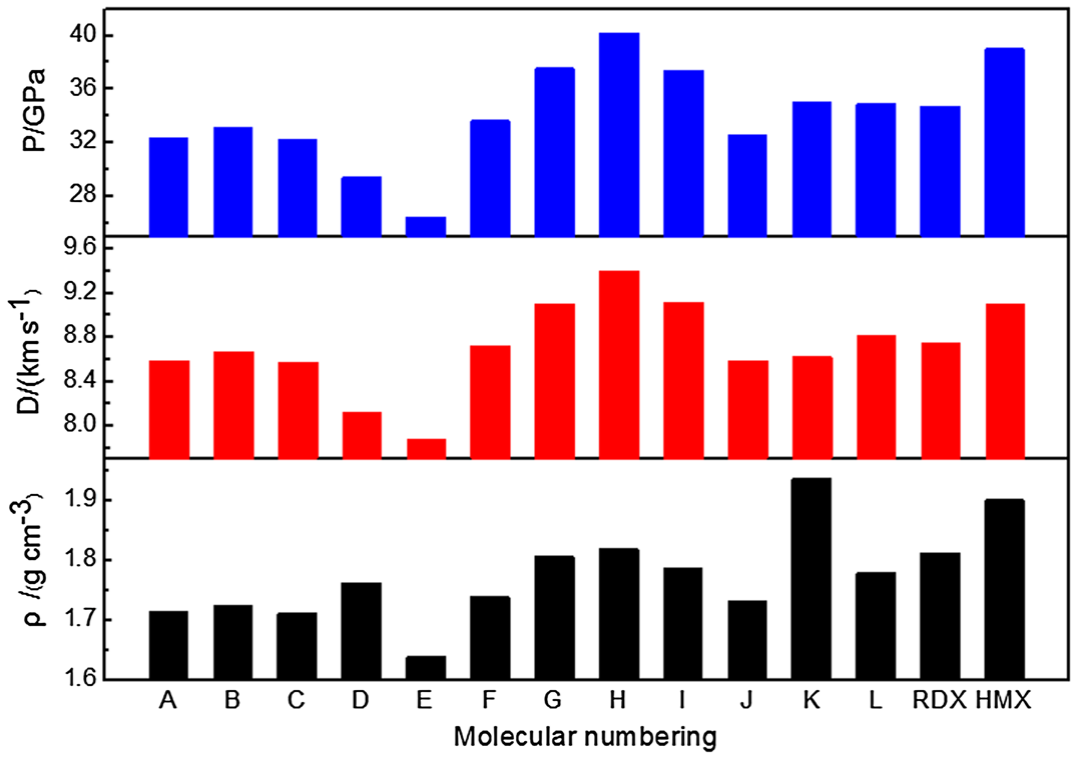
Comparison of the densities, detonation velocities and detonation pressures for1,3-dinitrohexahydropyrimidine-based compounds.

Table 3 presents the molecular weights (*M*_W_), OB(CO_2_) and the predicted densities (*ρ*), heats of detonation (*Q*), detonation velocities (*D*) and detonation pressures (*P*) of 1,3-dinitrohexahydropyrimidine and its derivatives along with cyclotrimethylenetrinitramine (RDX) and cyclotetramethylene tetranitramine (HMX). Figure 5 illustrates a comparison of energetic properties for the title compounds. As shown in Table 3, the calculated density and detonation velocity of A, RDX and HMX are approximately close to the measured values in literature^10^, demonstrating the reliability of the calculation method to some extent. According to Kamlet–Jacobs equation^42^, *ρ* is a key factor to influence *D* and *P*. Thus, density is one of the most important physical properties for all EMs. It is found that the introduction of the detonating groups into hexahydropyrimidine ring makes a significant increase of density compared to the unsubstituted compound (S). However, when incorporating –N_3_, there is an unexpectedly little increase of *ρ*. The possible reason is that –N_3_ group contributes a little to the mass of molecule but enhances the volume markedly and reduces the packing regularity on the other hand. One should note that all the disubstituted (attached to C atom) compounds possess higher densities than corresponding monosubstituted ones. Particularly, K with two –NF_2_ substitution groups features such good density that even higher than 1.93 g/cm^3^ when compared to RDX (1.816 g/cm^3^) and HMX (1.902 g/cm^3^).

**Table 3:** The molecular weights (*M*_W_), OB(CO_2_) and the predicted densities (*ρ*), heats of detonation (*Q*), detonation velocities (*D*) and detonation pressures (*P*) of the title compounds along with RDX and HMX.

Compd	Formula	*M*_W_	OB(CO_2_)/%	*ρ*/(g cm^−3^)	*Q*/(kJ kg^−1^)	*D*/(km s^−1^)	*P*/GPa
S	C_4_H_8_N_4_O_4_	176.16	− 72.66	1.595	6505.91	7.73	25.07
A	C_4_H_7_N_5_O_6_	221.15	− 39.79	1.715 (1.725)^a^	7193.16	8.58 (8.02)^a^	32.34
B	C_4_H_7_N_5_O_7_	237.15	− 30.36	1.725	7115.27	8.67	33.15
C	C_4_H_8_N_6_O_6_	236.17	− 40.65	1.713	6984.18	8.57	32.25
D	C_4_H_7_N_5_O_4_F_2_	227.15	− 52.83	1.764	5894.04	8.12	29.42
E	C_4_H_7_N_7_O_4_	217.17	− 55.26	1.640	6068.65	7.87	26.45
F	C_5_H_6_N_6_O_8_	278.17	− 28.76	1.740	7527.52	8.72	33.65
G	C_5_H_7_N_6_O_8_F	298.17	− 29.51	1.807	7943.06	9.10	37.56
H	C_5_H_7_N_7_O_10_	325.18	− 17.22	1.820	8284.03	9.40	40.20
I	C_4_H_6_N_6_O_8_	266.15	− 18.03	1.787	7640.44	9.12	37.40
J	C_4_H_6_N_8_O_6_	262.17	− 30.52	1.734	6787.83	8.59	32.60
K	C_4_H_6_N_6_O_4_F_4_	278.15	− 40.27	1.937	5701.81	8.62	35.08
L	C_4_H_6_N_5_O_6_F	239.14	− 33.45	1.780	7321.31	8.81	34.88
RDX	C_3_H_6_N_6_O_6_	222.14	− 21.61	1.786 (1.816^c^)	6279.87	8.76 (8.75^c^)	34.57 (34.70^c^)
HMX	C_4_H_8_N_8_O_8_	296.18	− 21.61	1.894 (1.902^c^)	6271.54	9.13 (9.10^c^)	38.85 (39.00^c^)

^a^Data in parentheses are from Ref.^10^.

^b,c^Experimental data from Refs.^58–60^.

As can be seen from Table 3, the monosubstitution of –CH(NO_2_)_2_, –CF(NO_2_)_2_ or –C(NO_2_)_3_ group and disubstitution of –NO_2_ groups (I) enhance OB values of the title compounds significantly, suggesting that OB is greatly influenced by the number of nitro groups. In addition, except for D, E and K, all the compounds have higher heats of detonation than those of RDX and HMX. Therein, H with –C(NO_2_)_3_ group attached to hexahydropyrimidine ring highlights the highest OB of -17.22 and largest Q of 8284.03 kJ kg^−1^.

The calculated detonation velocities of the title compounds lie in the range between 7.87–9.40 km s^−1^, and the calculated detonation pressures are between 26.45 GPa and 40.20 GPa. On the whole, –NO_2_, –ONO_2_, –NF_2_, –N_3_, –CH(NO_2_)_2_, –CF(NO_2_)_2_ and –C(NO_2_)_3_ are effective structural units to improve the detonation performance of 1,3-dinitrohexahydropyrimidine compounds. There is a particular increase in the *D* and *P* values when incorporating –CF(NO_2_)_2_ or –C(NO_2_)_3_ group. It is worth noting that the disubstituted compounds own higher *D* and *P* values than corresponding monosubstituted ones, suggesting that the increase of the energetic groups on hexahydropyrimidine ring benefits for desirable energetic performance. From Fig. 5, it can be find that the *D* and *P* values of G, H and I are very high and close to or above those of RDX. Meanwhile, H stands out among all the compounds with detonation velocity of 9.40 km s^−1^ and detonation pressure of 40.20 GPa, even higher than those of HMX. Although the heats of formation of D and K are not outstanding, their high densities compensate the disadvantage, proving again that the detonation performance of an energetic material is affected by both density and heat of formation.

### Thermal stability

**Figure 6:**
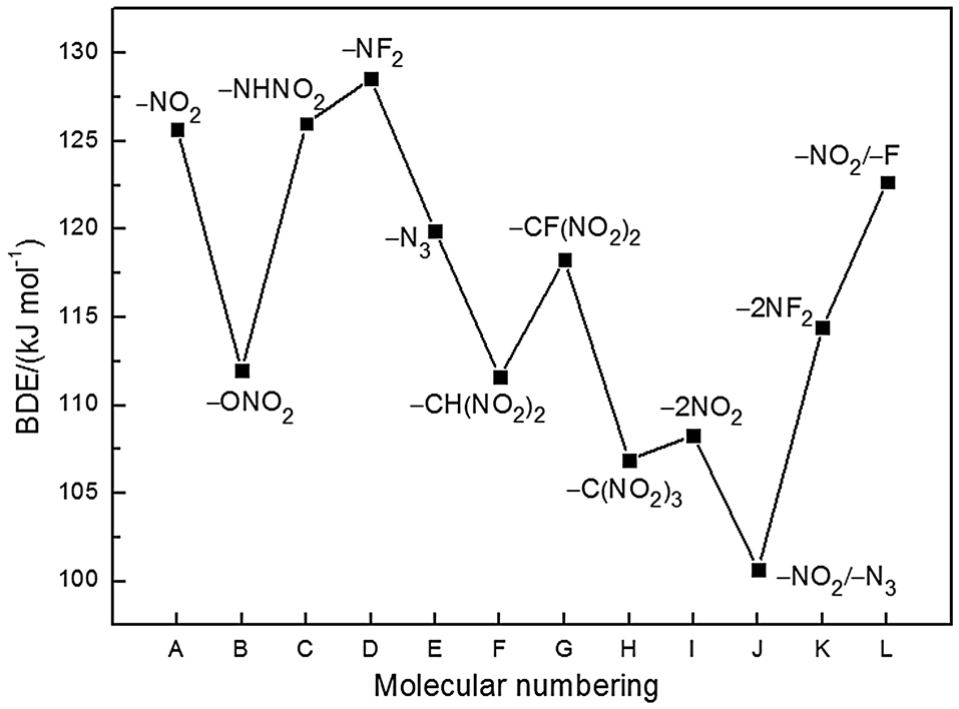
Bond dissociation energies (BDE) of 1,3-dinitrohexahydropyrimidine-based compounds.

Bond dissociation energy (BDE) provides useful information for understanding the thermal stability of the title compounds. To elucidate the pyrolysis mechanism and thermal stability of the title compounds, the BDEs of the relatively weak bonds (ring N–NO_2_, C–R or N–R') were calculated and the results were listed in Supplementary Table S3. A comparison of BDEs of the title compounds is displayed in Fig. 6. For monosubstituted compounds (except B and H) and disubstituted compounds J and L, the BDE of ring N–NO_2_ bond is much smaller than that of the C–R or N–R' bond, which shows that the ring N–NO_2_ bond cleavage is a possible thermal decomposition path for these compounds. It is the O–NO_2_ bond of the substituent –ONO_2_ group in B and C–NO_2_ bond of –C(NO_2_)_3_ group in H that signify the trigger bond in the compounds. Besides, it is found that the introduction of the substituent(s) onto hexahydropyrimidine ring weakens the N–NO_2_ bond with decreased BDE values (except B) when compared to S. In principle, all the compounds are energetic materials with BDE > 80 kJ mol^−1^ and A, C, D and L suffice the stability requirements of high energy density materials with BDE values over 120 kJ mol^−1^. On the contrary, the substitution of –ONO_2_, –N_3_, –CH(NO_2_)_2_, –CF(NO_2_)_2_, or –C(NO_2_)_3_ group lowers the stability of the compounds. Meanwhile, the BDEs of disubstituted (attached to C atom) hexahydropyrimidine compounds are lower than those of monosubstituted ones, namely increasing the number of energetic substituents is unfavorable with a view to the thermal stability. However, one should note that the BDEs are simply one piece of evidence for molecular thermal stability, the mechanism for the pyrolysis of compounds is mainly linked to their molecular structure.

### Impact sensitivity

The impact sensitivity of the title compounds are analyzed from several aspects: the Mulliken net charges of nitro group (*Q*_NO2_), electrostatic potential maxima (*V*_s,max_) and characteristic height (*H*_50_), and the results are presented in Table 4. Firstly, Zhang^44^ pointed out that the more negative charges the nitro group has, the more stable and insensitive the compound is. It can be seen that the incorporation of the substituents at C5- position induces less negative charges of the nitro groups and higher sensitivity than the parent compound (S). It is found that the disubstituted compounds possess less negative charges than corresponding monosubstituted ones, which gives the conclusion that the increase in the number of detonating groups on hexahydropyrimidine ring is unfavorable in view of the impact sensitivity. However, it is encouraging to find that the number of –NF_2_ group acts little on the negative charges of nitro group when compared D with K. It is verified from the nitro group charge calculations that –CH(NO_2_)_2_, –CF(NO_2_)_2_ and –C(NO_2_)_3_ groups are energetic explosopheres that often lead to higher sensitivity.

**Table 4:** Mulliken net charges of nitro group (*Q*_NO2_), electrostatic potential maxima (*V*_s,max_) and characteristic height (*H*_50_) of the title compounds.

Compd	*Q*_NO2_	*V*_s,max_/(kcal mol^−1^)	*H*_50_/cm
S	− 0.1744	34.03	54.15
A	− 0.1305	42.45	36.77
B	− 0.1489	36.71	46.60
C	− 0.1387	49.18	34.23
D	− 0.1455	36.98	50.19
E	− 0.1280	41.60	34.10
F	− 0.1258	47.05	30.86
G	− 0.1355	43.44	27.24
H	− 0.1198	48.37	22.12
I	− 0.1245	47.67	30.51
J	− 0.1244	41.92	32.43
K	− 0.1445	36.38	48.49
L	− 0.1322	45.20	34.87
RDX	− 0.0898	46.99	27.47 (28^a^)
HMX	− 0.1167	50.22	29.89 (32^a^)

^a^Experimental data from Ref.^58^.

Secondly, electrostatic potential is an important element to take into consideration for analyzing sensitivity. It was first proposed by Politzer et al.^38^, extensively developed by later researchers that the impact sensitivity of explosives has a positive correlation with the surface potential maxima (*V*_s,max_), namely, the impact sensitivity increases with the more positive value of *V*_s,max_. As seen from Table 4, the –ONO_2_ and –NF_2_ derivatives exhibit lower *V*_s,max_ values than other derivatives, suggesting that –ONO_2_ and –NF_2_ groups are desirable insensitive groups to construct new molecule frameworks. However, the substitution of other energetic groups leads to positive charge accumulation and the repulsion between these positive potentials elevates the resistance to shear slide, which are detrimental to the stability of the explosives. Except for K with two –NF_2_ group, the increase of the substituents does harm to the stability of the title compounds.

In addition, the characteristic height (*H*_50_) was calculated for the title compounds. It is known that the greater *H*_50_ the compound has, the less sensitive the compound is. The calculated *H*_50_ values of RDX and HMX are approximate to the literature measured values, implying that the calculation method is to some extent reliable to predict the *H*_50_ values of nitramine explosives. It can be seen that B, D and K exhibit good stability with *H*_50_ higher than 45 cm, demonstrating that these compounds are impact insensitive to external stimuli relatively. Therein, D owns the highest *H*_50_ value of 50.19 cm and thereby the most stable among the derivatives. The incorporation of –CF(NO_2_)_2_ or –C(NO_2_)_3_ group is in fact detrimental to the stability as predicted above.

Overall, it is found that the conclusions from *Q*_NO2_, *V*_s,max_ and *H*_50_ analyses are consistent roughly despite of some discrepancy. –ONO_2_ and –NF_2_ substituted compounds possess lower impact sensitivity given the above analyses. The impact sensitivity of 1,3-dinitrohexahydropyrimidine compounds rises drastically with the increase of nitro groups. Therefore, one should be cautious in importing nitro groups in molecular design given the stability requirement.

### Potential candidates for HEDMs

**Figure 7:**
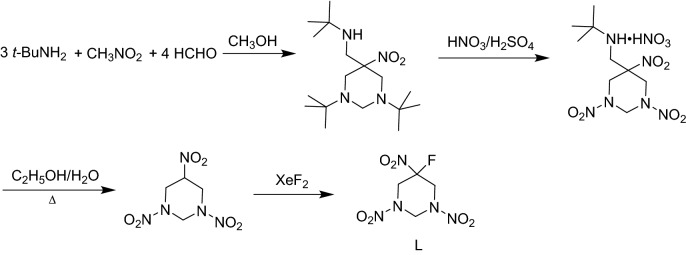
A possible synthetic route of L.

A potential candidate for HEDM should not only have excellent detonation properties, but also could exist stably. Most of hexahydropyrimidine derivatives possess desirable energetic performance, good thermal stability and low impact sensitivity. It is observed that the derivatives B, F, G, H, I, K and L have equivalent or higher detonation performance (*D*, *P*) than RDX. Besides, except for F, G, H and I, all the derivatives possess lower impact sensitivity than RDX. One should note that K and L feature high BDEs for the weakest bonds. On the basis of the above suggestions, K and L may be considered as the potential candidates of HEDMs, prompting further investigation in compound synthesis. Chapman et al*.*^13^ have reported a viable route to synthesize K by the reaction of ketone carbonyl groups with difluoramine or difluorosulfamic acid in the presence of a strong acid. A possible synthetic route was designed for L based on Mannich reaction and oxidation reactions as shown in Fig. 7:

Although A, I, J and K have been successfully synthesized, some detonation and thermodynamics properties are still lacking. In addition, the syntheses of other energetic compounds have not been reported yet. Thus, further investigations are still needed.

In summary, the introduction of the substituents onto hexahydropyrimidine ring leads to some significant structure deformation and distortion compared to 1,3-dinitrohexahydropyrimidine. –NO_2_, –ONO_2_, –NF_2_, –N_3_, –CH(NO_2_)_2_, –CF(NO_2_)_2_ and –C(NO_2_)_3_ are effective structural units to improve the detonation performance of 1,3-dinitrohexahydropyrimidine-based compounds. As mentioned above, the molecule structure is a decisive factor for the stability of a nitramine explosive. For most of the title compounds, N–NO_2_ bonds possess the least bond dissociation energy and may be the trigger bonds in these compounds. The substitution of –NHNO_2_, –NF_2_ or –F shows relatively higher BDE value and better thermal stability among all the derivatives. The impact sensitivity of the title compounds increases with the increasing number of nitro groups in molecules. The Mulliken net charges of nitro group (*Q*_NO2_), electrostatic potential maxima (*V*_s,max_) and characteristic height (*H*_50_) are significant parameters to evaluate the sensitivity of nitramine explosives. As the factors affecting the impact sensitivity are complicated, these elements should be considered as more to predict the impact sensitivity of an energetic material comprehensively.

## Conclusions

In the present study, the geometric and electronic structures, HOFs, energetic properties, thermal stability and impact sensitivity of 1,3-dinitrohexahydropyrimidine derivatives were analyzed. The results show that the substitution of –N_3_, –NO_2_, –NHNO_2_, –CH(NO_2_)_2_, –CF(NO_2_)_2_ or –C(NO_2_)_3_ group contributes positively to the HOFs of the derivatives. Besides, these groups together with –ONO_2_ and –NF_2_ are effective structural units to improve the detonation performance. Therein, H features the highest HOF and best detonation performance way above HMX.

The calculated frontier orbital energy shows that H may be the most reactive and K the least reactive among these compounds. An analysis of the bond dissociation energies for the weakest bonds reveals that for most 1,3-dinitrohexahydropyrimidine- based compounds, the ring N–NO_2_ bond cleavage is a possible thermal decomposition path for these compounds.

It is found that –ONO_2_ and –NF_2_ are effective energetic groups to construct impact insensitive frameworks. Therein, D owns the highest *H*_50_ value of 50.19 cm. Taking into consideration these results, it is regarded that K and L may be the potential candidates of HEDMs with powerful energetic performance and low sensitivity. It is anticipated that these findings will enhance the future prospects for rational energetic materials design.

## Computational method

DFT-B3LYP has been shown to accurately predict the structural parameters and perform frequency calculations on systems containing C, H, O, N and other elements in the study of energetic materials according to a series of studies by Xiao Heming et al.^45–47^. The geometry optimization and frequency analysis of the title compounds were fully performed at B3LYP/6-311G(d,p) level using density functional theory (DFT) embedded in Gaussian 09 program^48^. The vibration frequency and infrared spectra (IR) analyses (Supplementary Figure S2) showed that none of the optimized structures exhibited imaginary frequencies. When all the optimized structures corresponded to the local energy minimum points on the respective potential energy surfaces, the stable structures were obtained. Quantitative analysis of molecular van der Waals (vdW) surface was carried out with Multiwfn program. The 0.001 isosurface of electron density was regarded as vdW surface, since this definition reflects specific electron structure features of a molecule, such as lone pairs, π electrons etc., this is also what the definition used in our analyses. The analysis of ESP on vdW surface has been further quantified to extract more information. Researchers have defined a set of molecular descriptors based on ESP on vdW surface, which are taken as independent variables of general interaction properties function (GIPF)^38,40,49^. GIPF successfully connects distribution of ESP on vdW surface and many condensed phase properties, including density, heat of sublimation, impact sensitivity and so on, which are detailed in the text. Besides, the HOF and BDE were calculated and discussed based on the optimized structure. The detonation properties were further estimated based on calculated density and HOF according to Kamlet–Jacobs equations.

The strength of bonding, which could be evaluated by bond dissociation energy (BDE), is fundamental to understand pyrolysis mechanism and the thermal stability of the compound^50,51^. The BDE of the molecule corresponding to the enthalpy of reaction A–B(g) → A·(g) + B·(g), which is required for homolytic bond cleavage at 0 K and 1 atm, was calculated in terms of Eq. (8)^52^:

8 $$BDE_{0} (A - B) = E_{0} \left( {A \cdot } \right) + E_{0} \left( {B \cdot } \right) - E_{0} (A - B)$$

where *E*_0_ is the total electronic energy of the species calculated at B3LYP/6-311G(d,p) level. One should note that DFT/B3LYP method has been found to reasonably predict accurate BDEs.

The bond dissociation energy with zero-point energy (ZPE) correction can be determined by Eq. (9)^18^:

9 $$BDE\left( {A - B} \right)_{ZPE} = BDE_{0} \left( {A - B} \right) + \Delta E_{ZPE}$$

where Δ*E*_ZPE_ is the difference between ZPEs of the products and reactants.

The Mulliken net charges of nitro group (*Q*_NO2_) is calculated by the sum of atomic charges on the nitrogen and oxygen atoms of the nitro group as follows^53,54^:

10 $$Q_{{NO_{2} }} = Q_{N} + Q_{O1} + Q_{O2}$$

Experimentally, impact sensitivity is characterized through a drop weight test. The drop height (*H*_50_) is defined as the height from which there is a 50% probability of initiating explosion^55,56^. Pospíšil et al. have proposed an empirical formula relating *H*_50_ to the electrostatic potential of an energetic molecule, as given by the following equation^57^:

11 $$H_{50} = \alpha_{2} \sigma_{ + }^{2} + \beta_{2} \nu + \gamma_{2}$$

where *ν* describes the degree of balance between positive and negative potential on the isosurface, and *σ*_+_ is the electrostatic potential for the positive charge. The values of the coefficients *α*_2_, *β*_2_ and *γ*_2_ are taken from Ref.^57^.

## Supplementary information

Supplementary Information.
