# Supplementary material for: Screening for energetic compounds based on 1,3-dinitrohexahydropyrimidine skeleton and 5-various explosopheres: molecular design and computational study
Source: Sci Rep. 2020 Oct 26;10:18292. doi: 10.1038/s41598-020-75281-5 (PMC7589465; doi:10.1038/s41598-020-75281-5)
Supplement: Supplementary file 1 — Supplementary Information. [file 41598_2020_75281_MOESM1_ESM.pdf]

Supporting Information

Screening for energetic compounds based on 1,3-dinitrohexahydropyrimidine skeleton and 5-various explosopheres: Molecular design and computational study

Binghui Duan [a], Ning Liu [a,b,*], Xianming Lu [a,b], Hongchang Mo [a], Qian Zhang [a], Yingzhe Liu [a,b], Bozhou Wang [a,b,*]

[a] Xi'an Modern Chemistry Research Institute, Xi'an 710065 (P. R. China)
[b] State Key Laboratory of Fluorine & Nitrogen Chemicals, Xi'an 710065 (P. R. China)

Email: flackliu@sina.com; wbz600@163.com

Table of contents

Figure S1 The optimized geometries and atomic numbering of 1,3-dinitrohexahydropyrimidine-based compounds

NBO Analysis

Table S1 The second-order perturbation energies $E^{(2)}$ (kJ mol^{-1}) corresponding to the most important charge transfer interactions (donor→acceptor) in the compounds studied by using B3LYP method

Table S2 Calculated gas-phase HOFs ($\Delta H_{f,gas}$), heats of sublimation (ΔH_{sub}) and solid-phase HOFs ($\Delta H_{f,solid}$) of 1,3-dinitrohexahydropyrimidine (S) and its derivatives

Table S3 Bond dissociation energy (BDE, kJ mol^{-1}) of the relatively weak bonds of the title compounds

Figure S2 The calculated IR spectra of 1,3-dinitrohexahydropyrimidine and its derivatives

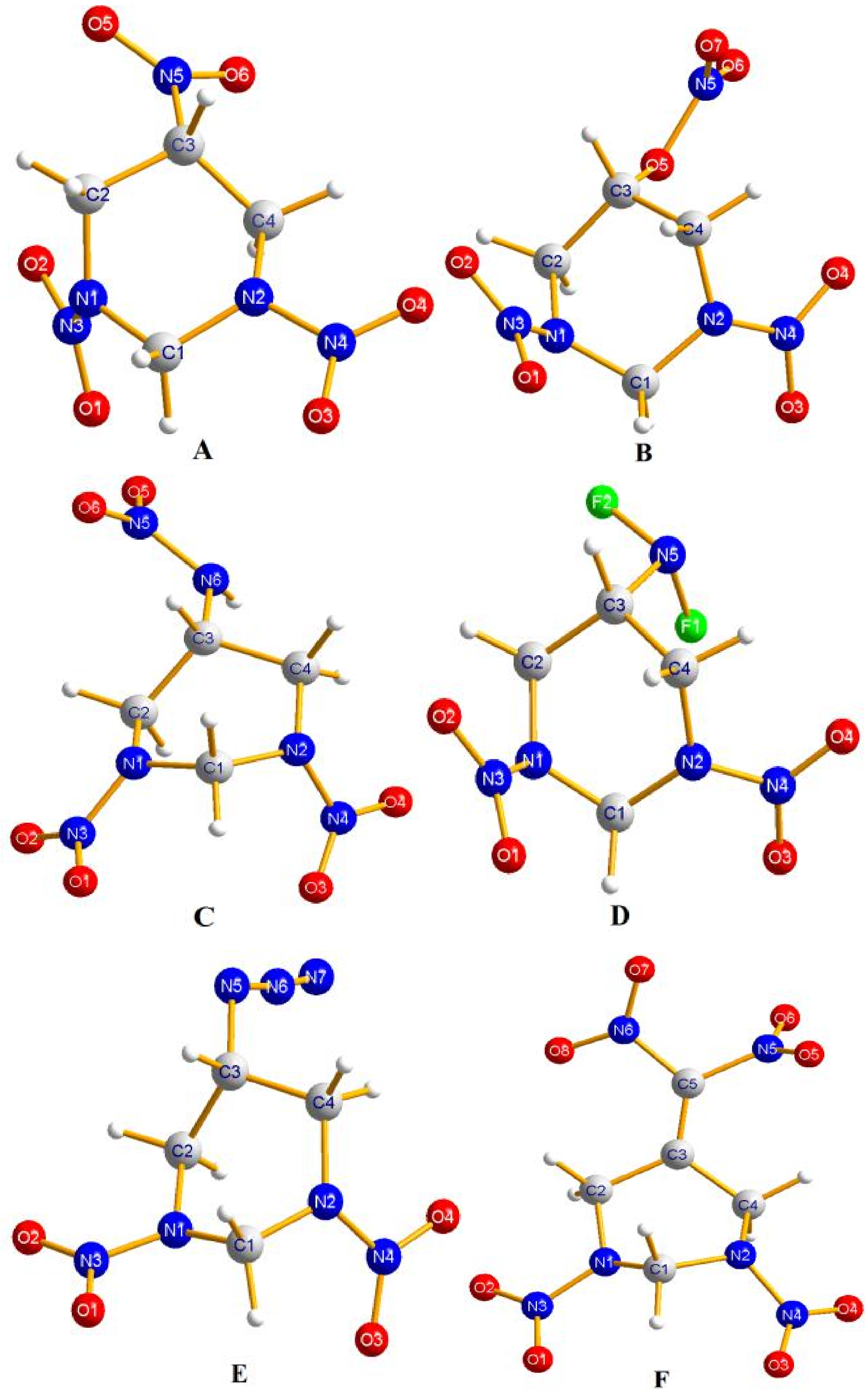
O5 N5 O6 C3 C2 C4 O2 N1 N3 N2 N4 O4 C1 O1 O3
A
O7 O6 N5 O5 C3 C4 C2 O2 N3 N1 N2 N4 O4 O1 C1 O3
B
O6 O5 N5 N6 C3 C4 C2 N1 C1 N2 O2 N3 O1 N4 O4 O3
C
F2 N5 C3 F1 C2 C4 O2 N1 N3 N2 N4 O4 C1 O1 O3
D
N5 N6 N7 C3 C4 C2 N2 O2 N1 N3 C1 N4 O4 O1 O3
E
O7 N6 O8 O6 N5 O5 C5 C3 C2 C4 N1 C1 N2 O2 N3 N4 O4 O1 O3
F

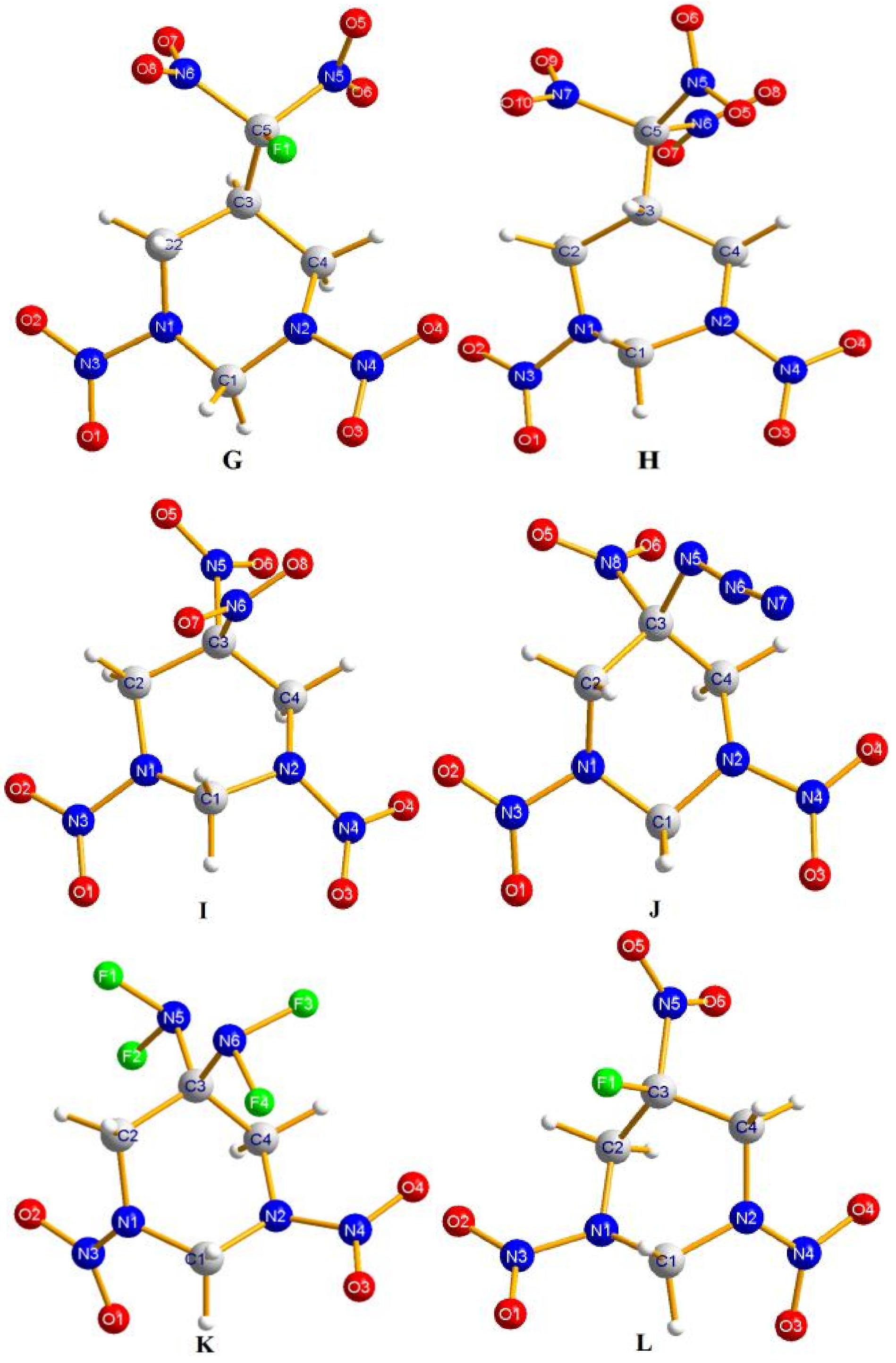

Note: Blue represents nitrogen atoms, gray represents carbon atoms, red represents oxygen atoms and green represents fluorine atoms.

Figure S1 The optimized geometries and atomic numbering of 1,3-dinitrohexahydropyrimidine-based compounds

NBO Analysis

NBO analysis has proved to be an effective tool for interpretation of hyperconjugative interaction. Thus, donor-acceptor (bond-antibond) interactions are taken into consideration by examining possible interactions between "filled" (donor) Lewis-type NBOs and "empty" (acceptor) non-Lewis NBOs and then estimating their energies by

second-order perturbation theory. The most important interactions between “filled” (donor) Lewis-type NBOs and “empty” (acceptor) non-Lewis NBOs are reported in Table S1. It could be seen from Table S1 that $E^{(2)}$ for LP(1) N6 as donors and BD*(1) C5–H9 anti-bond as acceptors [LP(1) N6→BD*(1) C5–H9] implies intramolecular hydrogen bond, which may be one of the stabilization forces for hexahydropyrimidine ring. Except for B and E, stabilization energy values for the $n_O \rightarrow \sigma^*_{N–N}$ interaction between the O11(12) LP(2) electron and the N2–N10 anti-bond and $n_N \rightarrow \sigma^*_{C–N}$ interaction between the N17 LP(2) electron and the C4–N16 anti-bond indicate that interactions mainly occur on the substituent groups between the lone pair of the O(N) and the N–NO_2 or C–R anti-bond. It could be deduced that interactions between the hexahydropyrimidine ring and the substituent groups are very strong. Taken together, these results indicate that hexahydropyrimidine derivatives have stable molecular structures.

Table S1 The second-order perturbation energies $E^{(2)}$ (kJ mol^{-1}) corresponding to the most important charge transfer interactions (donor → acceptor) in the compounds studied by using B3LYP method

Compd.	Don.NBO	Acc.NBO	$E^{(2)}$	Compd.	Don.NBO	Acc.NBO	$E^{(2)}$
A	LP(1) N6	BD*(1) C5–H9	6.55	G	LP(1) N6	BD*(1) C5–H9	6.15
	LP(2) O11	BD*(1) N2–N10	23.52		LP(2) O12	BD*(1) N2–N10	22.09
	LP(2) O15	BD*(1) N6–N13	21.45		LP(2) O15	BD*(1) N6–N13	21.92
	LP(2) O17	BD*(1) C4–N16	14.86		LP(2) F20	BD*(1) C4–C19	7.07
B	LP(1) N6	BD*(1) C1–H8	5.25	H	LP(1) N2	BD*(1) C1–H8	6.25
	LP(2) O11	BD*(1) N2–N10	22.75		LP(2) O11	BD*(1) N2–N10	22.03
	LP(2) O15	BD*(1) N6–N13	21.97		LP(2) O22	BD*(1) C20–N21	20.25
	LP(2) O22	BD*(1) O20–N21	38.18		LP(2) O28	BD*(1) C20–N27	21.44
	LP(1) O23	BD*(1) C4–O20	0.83		LP(2) O23	BD*(1) C4–C20	1.04
C	LP(1) N2	BD*(1) C3–H7	6.53	I	LP(1) N2	BD*(1) C3–H19	6.79
	LP(2) O11	BD*(1) N2–N10	23.02		LP(2) O11	BD*(1) N2–N10	22.73
	LP(2) O14	BD*(1) N6–N13	21.47		LP(2) O14	BD*(1) N6–N13	22.50
	LP(2) O21	BD*(1)C4–N23	1.09		LP(2) O23	BD*(1) C4–N22	18.12
D	LP(1) N6	BD*(1) C5–H18	5.57	J	LP(1) N2	BD*(1) C3–H17	6.46
	LP(2) O11	BD*(1) N2–N10	23.14		LP(2) O12	BD*(1) N2–N10	22.68
	LP(2) O15	BD*(1) N6–N13	21.47		LP(2) O15	BD*(1) N6–N13	22.43
	LP(2) F21	BD*(1) C4–N16	3.06		LP(2) O23	BD*(1) C4–N22	16.95
E	LP(1) N2	BD*(1) C1–H8	5.62	K	LP(1) N6	BD*(1) C5–H9	5.57
	LP(2) O12	BD*(1) N2–N10	23.33		LP(2) O11	BD*(1) N2–N10	23.98
	LP(2) O14	BD*(1) N6–N13	21.29		LP(2) O15	BD*(1) N6–N13	22.04
	LP(2) N16	BD*(2) N21–N22	125.82		LP(2) F21	BD*(1) C4–N16	3.44
F	LP(1) N2	BD*(1) C3–H16	6.39	L	LP(1) N6	BD*(1) C5–H20	5.35
	LP(2) O11	BD*(1) N2–N10	22.99		LP(2) O12	BD*(1) N2–N10	24.17
	LP(2) O14	BD*(1) N6–N13	22.45		LP(2) O14	BD*(1) N6–N13	22.25
	LP(2) O21	BD*(1) C19–N20	15.60		LP(2) O17	BD*(1) C4–N16	18.64
	LP(2) O24	BD*(1) C19–N23	15.73		LP(2) F22	BD*(1) C4–N16	14.64

Table S2 Calculated gas-phase HOFs ($\Delta H_{f,gas}$), heats of sublimation (ΔH_{sub}) and solid-phase HOFs ($\Delta H_{f,solid}$) of 1,3-dinitrohexahydropyrimidine (S) and its derivatives

Compd.	$\Delta H_{f,gas}$/kJ mol^{-1}	ΔH_{sub}/kJ mol^{-1}	$\Delta H_{f,solid}$/kJ mol^{-1}
S	159.072	90.518	178.554
A	349.529	97.218	252.312
B	300.190	101.017	152.173
C	397.729	109.303	288.426
D	279.145	99.203	169.942
E	447.400	104.427	372.973
F	498.616	114.196	384.420
G	604.956	119.492	485.464
H	700.797	132.545	568.252
I	429.189	105.131	324.058
J	574.043	110.430	463.613
K	336.673	106.428	230.245
L	393.142	97.329	295.813

Table S3 Bond dissociation energy (BDE, kJ mol^{-1}) of the relatively weak bonds of the title compounds

Compd.	C–NO_2	N–NO_2	C–R	N–R'
S		131.59		
A	162.64	125.68		
B		131.81	263.53(C–ONO_2)	111.98(O–NO_2)
C		126.01	288.87(C–$NHNO_2$)	
D		128.53	191.58(C–NF_2)	
E		119.90	210.10(C–N_3)	
F	193.94	111.63		
G	131.64	118.25	407.77(C–F)	
H	106.91	119.06		
I	108.28	115.30		
J	102.35	100.69	185.38(C–N_3)	
K		124.83	114.42(C–NF_2)	
L	174.21	122.69	392.13(C–F)	

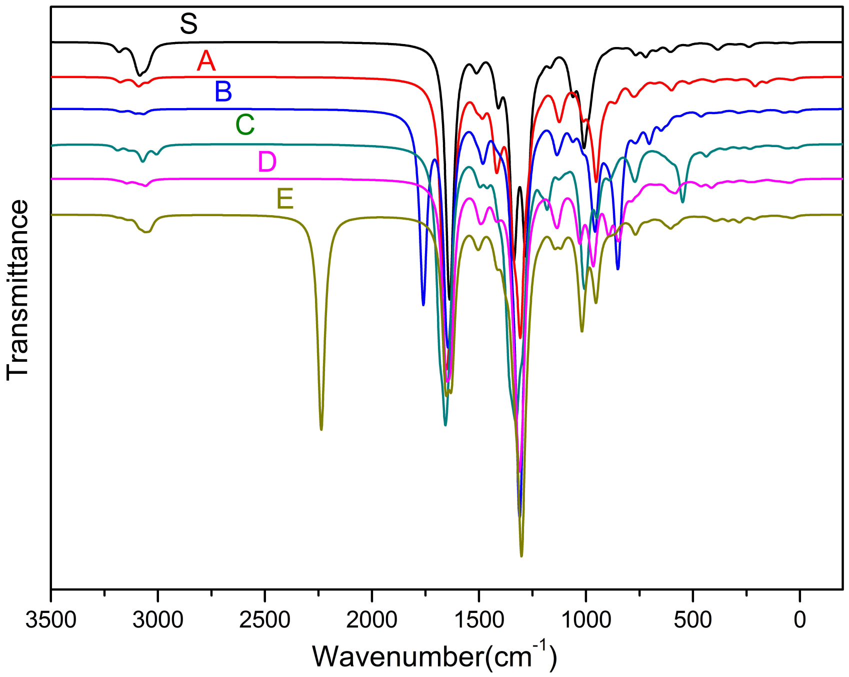

(a)

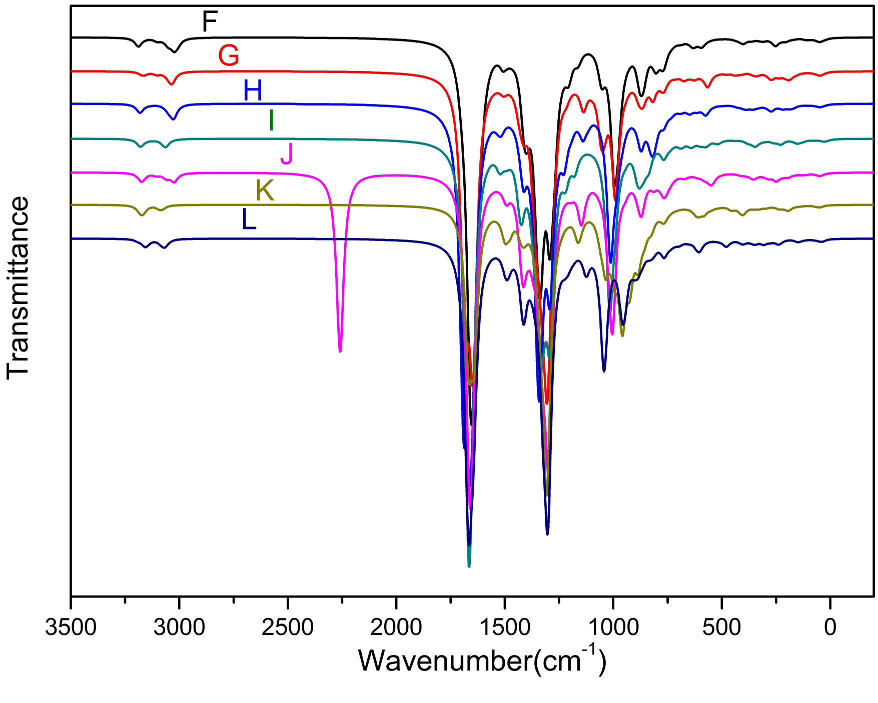

(b)

Figure S2 The calculated IR spectra of (a) 1,3-dinitrohexahydropyrimidine(S) and its derivatives A~E; (b) F~L.

From Figure S2, it is clear that there are two strong $–NO_2$ stretching vibration absorption peaks around 1650 and 1300 cm^{-1}, respectively. A series of small peaks at 920~850 cm^{-1} corresponds to the stretching vibration peaks of C–N. The characteristic absorption peak of $–N_3$ at 2200 cm^{-1} were identified for E and J. The absorption peak around 1000 cm^{-1} of G and L could be assigned to the characteristic absorption peak of C–F. The calculated IR spectra of the title compounds are expected to serve as a

reference for the synthetic structure verification of these compounds.
